# Indicators of Cardiometabolic Function in Pregnancy and Long-Term Risk of COVID-19: Population-Based Cohort Study

**DOI:** 10.7759/cureus.35325

**Published:** 2023-02-22

**Authors:** Joel G Ray, Eyal Cohen, Emily Ana Butler, Sonia Grandi, Alison Park

**Affiliations:** 1 Obstetrical Medicine, University of Toronto, Toronto, CAN; 2 Medicine and Obstetrics, St. Michael's Hospital, Toronto, CAN; 3 Pediatrics, The Hospital for Sick Children, Toronto, CAN; 4 Pediatrics, University of Toronto, Toronto, CAN; 5 Epidemiology, Institute for Clinical Evaluative Sciences (ICES), Toronto, CAN

**Keywords:** obstetrics, pregnancy, hyperglycemia, metabolic syndrome, prematurity, body mass index, covid-19, sars-cov-2

## Abstract

Background: Pregnancy increases a woman’s susceptibility to severe COVID-19, especially those with metabolic dysfunction. It is unknown if markers of metabolic dysfunction commonly assessed around pregnancy are associated with COVID-19 illness after pregnancy.

Aim: The aim of this study is to evaluate the indicators of metabolic dysfunction collected in pregnancy and the future risk of severe COVID-19 after pregnancy.

Methods: This population-based cohort study was completed in all of Ontario, comprising 417,713 women aged 15-49 years with a hospital birth between April 2007 and March 2018. The main exposure was each 1-kg/m^2^ higher body mass index (BMI), 1-mmol/L higher glucose concentration at the 50-g glucose challenge test, and one-week earlier gestational week at delivery. The main outcome was severe COVID-19 illness or death, from the start of the pandemic period on March 1, 2020, till December 31, 2021.

Results: The adjusted hazard ratio (aHR) of COVID-19 illness increased per 1-kg/m^2^ higher BMI (1.05, 95% CI 1.04-1.06), per 1-mmol/L higher serum glucose concentration (1.16, 95% CI 1.10-1.22), and for each one-week earlier gestational week at delivery (1.12, 95% CI 1.03-1.23). Relative to women with no dichotomized risk factors, the aHR for severe COVID-19 was 1.60 (95% CI 1.28-2.01) with one factor, 3.34 (95% CI 2.51-4.44) with two factors, and 4.52 (95% CI 2.11-9.67) with three factors.

Conclusions: The number, and degree, of standard metabolic indicators measured around pregnancy predict the future risk of severe COVID-19 remotely after that pregnancy.

## Introduction

Gestational diabetes mellitus (DM), higher body mass index (BMI) in pregnancy, and preterm delivery partly predict the onset of metabolic syndrome (MetSyn) in women years after pregnancy [1,2]. After the emergence of the SARS-COV-2 pandemic around March 2020, it became evident that pregnant women were prone to severe COVID-19 illness and adverse perinatal outcomes, such as preterm labor and preterm birth [3]. The risk of adversity was most pronounced in women with a high BMI or gestational DM within the index pregnancy [4]. What is not known, however, is whether the aforementioned factors, when measured in pregnancy, are associated with the onset of COVID-19 illness well after that pregnancy has ended. The availability of three standardized continuous measures, namely, pre-pregnancy BMI, glucose concentration at the time of gestational DM screening, and gestational week at delivery, enabled us to address this question.

This study evaluated the future risk of severe COVID-19 in relation to prior pregnancy BMI, serum glucose concentration, and gestational age at birth - both as continuous and dichotomized risk factors.

## Materials and methods

This study considered all women aged 15-49 years with a hospital live birth or stillbirth in Ontario, Canada, from April 1, 2007, to March 31, 2018. Ontario has universal healthcare under the Ontario Health Insurance Plan (OHIP), and most women undergo gestational DM screening with a 50-g glucose challenge test (GCT) at about 27 weeks of gestation (Appendix table).

All women with known pre-pregnancy BMI and a GCT result in the index pregnancy were included. Those with pre-pregnancy DM and women not alive or eligible for OHIP on March 1, 2020 (the start of the SARS-CoV-2 pandemic) were excluded. If a woman had more than one eligible delivery during the study period, then her latest birth was considered. The last birth was considered up to March 31, 2018, to minimize the chance that a woman was pregnant, or had recently given birth, at the onset of the COVID-19 pandemic and because BMI data were only available up to that date.

Births and outpatient and inpatient encounters were captured in province-wide administrative datasets that were linked using unique encoded identifiers and analyzed at ICES (https://datadictionary.ices.on.ca/Applications/DataDictionary/Default.aspx), as described by Catov et al. and Li et at. [1,5] and in Appendix table. BMI was identified in the Better Outcomes Registry and Network (BORN) Information System and Niday Perinatal Databases with a 50-g GCT in the Ontario Laboratory Information System [6]. All SARS-CoV-2 vaccinations in Ontario are also captured at ICES (https://data.ontario.ca/dataset/covid-19-vaccine-data-in-ontario). More than 95% of pregnancies have an ultrasound enabling accurate pregnancy dating [7].

The main study outcome was severe COVID-19 illness arising from the start of the pandemic period of March 1, 2020 (i.e., time zero), to December 31, 2021 (the latest complete data). Severe COVID-19 illness was based on a positive SARS-CoV-2 PCR test within seven days preceding, or up to three days after, hospitalization or death [6].

Analyses

Time-to-event analyses started at time zero. Separate Cox proportional hazard models generated hazard ratios (HRs) and 95% CI for the respective relationships between the study outcomes and each 1-kg/m^2^ incremental higher BMI; 1-mmol/L higher GCT glucose concentration, and one-week earlier gestational week at delivery, from ≥37 weeks (term birth) declining weekly down to ≤24 weeks. Next, each study outcome was assessed about having 0 (referent), 1, 2, or 3 dichotomized risk factors (i.e., BMI ≥ 30 kg/m^2^, positive 50-g GCT ≥ 7.8 mmol/L, and/or preterm birth < 37 weeks).

HRs were adjusted for age, rural residence, and area income quintile at the index birth; a woman’s age at time zero; chronic hypertension in the index pregnancy or up to two years before that pregnancy; and the time-varying SARS-CoV-2 first vaccination date. Censoring was based on death occurring prior to either outcome, loss of OHIP eligibility, or arrival at the end of the study period of December 31, 2021. A further analysis was censored at the start of any subsequent pregnancy during the SARS-CoV-2 pandemic.

Sample size estimations were not performed as the current study used a fixed population-based data sample.

Ethics approval

Datasets were linked using unique encoded identifiers and analyzed at ICES. The use of data in this project was authorized under section 45 of Ontario’s Personal Health Information Protection Act, which does not require the approval of a research ethics board.

## Results

A total of 417,713 women were included, followed by a median (IQR) of 1.8 (1.8, 1.8) years after time zero, totaling 756,471 person-years of follow-up. Their mean (SD) age at the index delivery was 31.1 (5.2) years, and 83.4% received at least one SARS-CoV-2 vaccination during the follow-up period (Table 1).

**Table 1:** Characteristics of 417,713 included women who had a live birth or stillbirth delivery between April 2007 and March 2018, and during the SARS-CoV-2 pandemic from March 1, 2020, to December 31, 2021. All data are presented as numbers (%) unless otherwise indicated. ^a^Measured at a mean (SD) gestational age of 26.8 (1.9) weeks. IQR: Interquartile range; SD: Standard deviation.

Characteristics	Measures
In the index delivery	
Mean (SD) age, y	31.1 (5.2)
Income quintile (Q)	
Q1 (lowest)	86,965 (20.8)
Q2	82,607 (19.8)
Q3	87,443 (20.9)
Q4	90,081 (21.6)
Q5 (highest)	69,744 (16.7)
Missing	873 (0.2)
Rural residence	36,000 (8.6)
Unknown residence	441 (0.1)
Median (IQR) parity	1 (0-1)
Multifetal pregnancy	7202 (1.7)
Chronic hypertension in the index pregnancy, or up to 2 years before	19,591 (4.7)
Stillbirth	831 (0.2)
Measures in the index pregnancy	
Mean (SD) pre-pregnancy body mass index, kg/m^2^	25.5 (6.2)
Mean (SD) glucose concentration at the 50-g glucose challenge test, mmol/L^a^	6.6 (1.8)
Mean (SD) gestational age at delivery, weeks	38.9 (1.6)
Preterm birth < 37 weeks of gestation	26,188 (6.3)
From the start of the COVID-19 pandemic on March 1, 2020 (time zero), to the end of the study follow-up on December 31, 2021	
Mean (SD) age, years	35.8 (5.6)
Any subsequent pregnancy	64,141 (15.4)
Median (IQR) no. of years of follow-up, from time zero	1.8 (1.8-1.8)
Received at least one SARS-CoV-2 vaccination	348,261 (83.4)

The adjusted HR of severe COVID-19 increased per 1-kg/m^2^ higher BMI (1.05, 95% CI 1.04-1.06), per 1-mmol/L higher serum glucose concentration (1.16, 95% CI 1.10-1.22), and for each one-week earlier gestational week at delivery (1.12, 95% CI 1.03-1.23) (Table 2).

**Table 2:** Pre-pregnancy body mass index, glucose concentration at the time of a 50-g glucose challenge test, and/or gestational age at delivery – each in association with severe COVID-19 disease well beyond pregnancy. Each individual risk factor (upper) as well as the number of these dichotomized risk factors combined (lower) is shown. Figures are shown for all 417,713 women included in the study cohort. ^a^Defined as a positive SARS-CoV-2 PCR test from seven days before and up to three days after hospitalization or seven days before or up to one day after death – arising from the start of the pandemic period on March 1, 2020 (i.e., time zero), to December 31, 2021. ^b^Adjusted for age, rural residence, and area income quintile – each at the index birth; chronic hypertension in the index pregnancy or up to two years before the pregnancy; a woman’s age on March 1, 2020; and the time-varying first SARS-CoV-2 vaccination date. Censoring is on a death occurring before either outcome, loss of OHIP eligibility, or arrival at the end of the study period. ^c^A total of 64,141 women (15.4%) were censored when they had another pregnancy during the SARS-CoV-2 pandemic, between March 1, 2020, and December 31, 2021, of whom 93 (1.5 per 1000) had severe COVID-19 in that period. ^d^Dichotomized risk factors are pre-pregnancy body mass index ≥ 30 kg/m^2^, positive 50-g glucose challenge test of ≥ 7.8 mmol/L, and/or preterm delivery < 37 weeks of gestation – each in the index pregnancy. CI: Confidence interval.

Risk factor assessed	Total no. of person-years of follow-up	No. of outcomes^a^ (rate per 1000 person-years)	Unadjusted hazard ratio (95% CI)	Adjusted hazard ratio (95% CI)^b^	Adjusted hazard ratio (95% CI)^b^ and further censoring on any future pregnancy^b,c^
Per 1-unit change in each risk factor					
Each 1-kg/m^2^ higher body mass index	756,471	-	1.06 (1.04 to 1.07)	1.05 (1.04 to 1.06)	1.06 (1.04 to 1.07)
Each 1-mmol/L higher serum glucose concentration	756,471	-	1.17 (1.12 to 1.23)	1.16 (1.10 to 1.22)	1.18 (1.12 to 1.25)
Each 1-week earlier gestational age at delivery	756,471	-	1.14 (1.05 to 1.24)	1.12 (1.03 to 1.23)	1.07 (0.95 to 1.20)
Number of dichotomized risk factors (number of women exposed)					
0 (N = 255,416)^d^	462,611	164 (0.4)	1.00 (referent)	1.00 (referent)	1.00 (referent)
1 (N = 130,157)^d^	235,691	141 (0.6)	1.69 (1.35 to 2.11)	1.60 (1.28 to 2.01)	1.53 (1.17 to 1.99)
2 (N = 30,019)^d^	54,334	70 (1.3)	3.64 (2.75 to 4.81)	3.34 (2.51 to 4.44)	3.57 (2.60 to 4.90)
3 (N = 2121)^d^	3835	7 (1.8)	5.16 (2.43 to 10.99)	4.52 (2.11 to 9.67)	4.70 (2.06 to 10.75)

Relative to women with no risk factors at their conventional cut points, the aHR for severe COVID-19 was 1.60 (95% CI 1.28-2.01) with one factor, 3.34 (95% CI 2.51-4.44) with two factors, and 4.52 (95% CI 2.11-9.67) in women with three factors (Table 2). There were 64,141 women (15.4%) who were pregnant during the SARS-CoV-2 pandemic, of whom 93 (1.5 per 1000) had severe COVID-19. Further censoring on pregnancy generated roughly the same aHRs (Table 2).

## Discussion

Elevated BMI and gestational DM are known risk factors for acquiring COVID-19 in pregnancy [4]. However, unlike prior studies, these two risk factors were handled here as continuous measures, and they were also assessed in relation to acquiring COVID-19 well beyond pregnancy. In a novel manner, gestational age at delivery was also assessed while avoiding any potential reverse causation introduced when COVID-19 and timing of birth were assessed in the same pregnancy because a severe infection may either precipitate or necessitate preterm birth [3].

The risk of severe COVID-19 was higher when each measure was analyzed continuously and even more so when combined together at conventional cut points. Recent data among middle-aged non-pregnant adults demonstrated a higher risk of COVID-19-related ICU admission, invasive mechanical ventilation, and mortality in the presence of the MetSyn [8]. While the current study could not formally assess all MetSyn criteria, maternal lipid profile, glucose intolerance in pregnancy, and pre-pregnancy BMI have been shown to predict the onset of MetSyn three months postpartum [9], as do a history of preterm delivery, gestational DM, and BMI years after pregnancy [1,2].

These study findings align with a body of work that considers in-pregnancy measures like BMI, glucose handling, and timing of delivery as a means to predict, and potentially modify, a woman’s future health. To date, studies have largely focused on future cardiometabolic health [9-11], but not new-onset infection. The current study further suggests that metabolic measures may offer a future perspective on a woman’s vulnerability to COVID-19. Even so, it remains to be determined whether these and other metabolic factors (e.g., blood pressure) influence her susceptibility to other types of viral and bacterial infections, or whether metabolic modification after birth can mitigate the onset of severe infectious illness.

Strengths and limitations

As a limitation, the current study adjusted for some confounders, such as rural residence and income level, but not race or ethnicity. Chronic hypertension was also accounted for, but blood pressure measures were not available. It is unlikely that caregiver burden can explain the relationship between preterm delivery and COVID-19 risk after pregnancy as parents of a preterm-born infant exhibit only slightly higher levels of stress than parents of term-born children [12]. Rather, women with the MetSyn are more likely to experience preterm delivery, especially provider-initiated preterm birth [13]. The study was conducted within a jurisdiction with relatively high vaccine uptake; so, the risk of severe COVID-19 may be even more pronounced in settings with lower vaccine use. While the first SARS-COV-2 vaccination was handled as a time-varying covariate, emerging SARS-CoV-2 variants were not differentiated.

## Conclusions

The risk of severe COVID-19 remotely after pregnancy is higher in the presence of metabolic indicators standardly measured around the time of pregnancy.

## Appendices

**Table 3:** Variables used to define cohort entry and exclusion criteria as well as study exposures, outcomes, and adjustment BIS: Better Outcomes Registry & Network (BORN) Information System; CIHI: Canadian Institute for Health Information; COVAXON: Ontario COVID-19 Vaccine Data; DAD: Discharge Abstract Database; ICD-9: International Classification of Diseases, 9th Revision; ICD-10-CA: International Classification of Diseases, 10th Revision, Canada; LOINC: Logical Observation Identifiers Names and Codes; NACRS: National Ambulatory Care Reporting System; ODD: Ontario Diabetes Dataset; OHIP: Ontario Health Insurance Plan; OLIS: Ontario Laboratories Information System; OLISC19: Ontario Laboratories Information System COVID-19 Laboratory Data; RPDB: Registered Persons Database; SDS: Same Day Surgery Database; MOMBABY: Mother-Baby database.

Assessment	Timing	Disease, procedure, or condition	CIHI-DAD, SDS, or NACRS ICD-10-CA diagnosis codes	OHIP ICD-9 diagnosis or fee codes (or other data sources, if in parentheses)	Validation studies or documentation for some codes
Cohort entry criterion	April 1, 2007, to March 31, 2018	Obstetrical delivery in the province of Ontario at ≥ 20 weeks of gestation	Main patient service code indicating “obstetrical delivery” (MOMBABY - includes linked CIHI-DAD inpatient admission records of delivering mothers and their infants)	-	-
Cohort exclusion criteria	At the index delivery	Invalid maternal healthcare number (HCN), sex, birth date, death date, or discharge date	-	Invalid HCN; or {RPDB} Birth date is missing or sex is missing or male; or index delivery discharge date or {RPDB} death date precedes the index admission date. The RPDB contains demographic information and encrypted healthcare numbers for all individuals eligible for OHIP.	-
	Same as above	Gestational age at delivery is missing or <20 weeks	Clinical gestation weeks at delivery in MOMBABY	-	-
	Same as above	Maternal age < 15 or > 49 years	-	{RPDB} age	-
	Same as above	Pre-pregnancy diabetes mellitus	-	{ODD}	-
	Same as above	Unknown pre-pregnancy body mass index	-	{BORN NIDAY or BIS}	
	Same as above	Unknown 50-g glucose challenge test at 23-32 weeks of gestation	-	{OLIS} 50-g glucose challenge test identified as follows: A. LOINC 14754-6; or B. LOINC 14756-1, when the same observation date has an observation with LOINC 4269-7 with a value of 50; or C. LOINC 14756-1, when there is no observation (or observation with a missing value) with LOINC 4269-7 on the same observation date, and there are no other glucose tests on the same observation date.	https://pubmed.ncbi.nlm.nih.gov/28507087/
	March 1, 2020 (time zero)	The woman was not a resident of Ontario at the start of the follow-up	-	{RPDB} Province number is not ‘35’	-
	Same as above	The woman was not alive at the start of the follow-up	-	{RPDB} Death date	-
	Same as above	The woman was not eligible for OHIP at the start of the follow-up	-	{RPDB} Start and end of eligibility	-
Main study exposures	In the index pregnancy	Pre-pregnancy body mass index, kg/m^2^	-	{BORN NIDAY or BORN BIS}	-
	Same as above	Serum glucose concentration at the 50-g glucose challenge test at 23-32 weeks of gestation	-	{OLIS} See Exclusions for 50-g glucose challenge test codes.	https://pubmed.ncbi.nlm.nih.gov/28507087/
	At the index delivery	Gestational age at delivery, in weeks	Clinical gestation weeks at delivery in MOMBABY	-	-
	In the index pregnancy or at the index delivery	Number of risk factors: (i) BMI ≥ 30 kg/m^2^; (ii) 50-g GCT ≥ 7.8 mmol/L; (iii) preterm birth < 37 weeks	See above	See above	-
Study outcome	March 1, 2020, to December 31, 2021, censoring at loss OHIP eligibility, end of the study period, or the day after death	Earliest SARS-CoV-2 positive test AND a hospital admission occurring up to 3 days before or 7 days after, or death up to 1 day before or 7 days after, the SARS-CoV-2 specimen date.	DAD (hospital admission, not alive at discharge), NACRS (hospital admission, not alive at discharge), SDS (not alive at discharge)	{OLISC19} SARS-CoV-2 test, {RPDB} Death date	For the ICES methodology and Python script for cleaning and parsing OLIS lab results for SARS-CoV-2 and other respiratory viruses, see https://github.com/icescentral/COVID19-Lab-Results.
Covariates	At the index delivery	Maternal age	-	{RPDB} Age	-
	Same as above	Area income quintile	-	{Statistics Canada Census}	-
	Same as above	Rural residence	-	{Statistics Canada Census}	-
	At the index delivery, or up to 2 years before the index pregnancy	Chronic hypertension	I10, I15, O10, O11	401	https://pubmed.ncbi.nlm.nih.gov/20101286/
	43891	Women’s age at the start of the follow-up	-	{RPDB} Age	-
	March 1, 2020, to December 24, 2021	COVID-19 first vaccination	-	{COVAXON} Date at 1^st^ vaccination	-
